# Molecular Epidemiological Survey of *Staphylococcus lugdunensis* Isolates With Variable Number of Repeats in the von Willebrand Factor-Binding Protein Gene

**DOI:** 10.3389/fcimb.2021.748640

**Published:** 2021-11-11

**Authors:** Lee-Chung Lin, Chun-Wen Cheng, Shih-Cheng Chang, Jang-Jih Lu

**Affiliations:** ^1^ Department of Laboratory Medicine, Chang Gung Memorial Hospital, Taoyuan, Taiwan; ^2^ Division of Infectious Diseases, Department of Internal Medicine, Chang Gung Memorial Hospital at Linkou, Chang Gung University College of Medicine, Taoyuan, Taiwan; ^3^ Department of Medical Biotechnology and Laboratory Science, College of Medicine, Chang Gung University, Taoyuan, Taiwan; ^4^ Department of Medicine, College of Medicine, Chang Gung University, Taoyuan, Taiwan

**Keywords:** *S. lugdunensis*, von Willebrand factor binding protein, pathogenesis, SCC*mec*, MLST

## Abstract

The von Willebrand factor binding protein in *Staphylococcus lugdunensis* (vWbl) comprises four major regions: the signal peptide (S), the non-repetitive (A) region, the repeat (R) region, and the wall-associated (W) region. Previous studies have demonstrated that the R region contains 10 copies of repeating sequences; however, we reveal that the copy number of repeats in the *vWbl* gene varies among different *S. lugdunensis* isolates. In this study, an epidemiological surveillance was conducted to determine whether the copy number of repeats in *vWbl* in different isolates of *S. lugdunensis* correlates with their infectivity. The number of repeats was estimated in a total of 212 isolates, consisting of 162 isolates of oxacillin-sensitive *S. lugdunensis* (OSSL) and 50 isolates of oxacillin-resistant *S. lugdunensis* (ORSL). Our data showed that 72.5% (116/162) of OSSL isolates contained 9 (25, 15.4%), 12 (43, 26.5%), or 13 (48, 29.6%) repeats, and 90% (45/50) of ORSL isolates had 9 (32, 64%) or 13 (13, 26%) repeats. In addition, 89.6% (26 of 29) of the sequence type (ST)27 strain had 12 repeats, and 86.8% (13 of 15) of the ST4 strain had 14 repeats. Twenty-seven of the 28 isolates with nine repeats were of the staphylococcal cassette chromosome *mec* (SCC*mec*) V or V_t_ type and belonged to ST3, and all isolates with 13 repeats were of SCC*mec* II type and belonged to ST6. All isolates with nine repeats had a stop codon at the 18^th^ codon of the third repeat, suggesting that these isolates coded for nonfunctional vWbl. Further, western blot analysis confirmed that all strains translated vWbl, and only vWbl proteins coded by genes with nine repeats were exported outside the cell. These results suggest that number of *vWbl* repeats in *S. lugdunensis* have clonal specificities and may correlate with potential pathogenicity.

## Introduction

*Staphylococcus lugdunensis* is a member of the coagulase-negative staphylococcal species (CoNS) and has been reported to cause a wide variety of diseases, such as skin and soft tissue infections, bone and joint infections, bacteremia, and infective endocarditis (Wu et al., 2011; Yeh et al., 2015; Douiri et al., 2016; Yeh et al., 2016). Previous studies have shown that the fatality rate of patients with endocarditis caused by an infection of *S. lugdunensis* is approximately 40% (Liu et al., 2010) and that of patients with endocarditis caused by an infection of other members of the CoNS is 14.3% (Fernández-Rufete et al., 2012; Molina et al., 2013), indicating that *S. lugdunensis* is more virulent than other members of the CoNS.

Previous studies have identified that the von Willebrand factor binding protein (vWbp), which is secreted by *S. aureus*, mediates the binding of *S. aureus* to platelets to form an aggregated complex (Bjerketorp et al., 2002). The vWbl share partially similarities with vWbp in *S. lugdunensis* (Nilsson et al., 2004), which was identified by an affinity selection study of a phage display library against the von Willebrand factor (vWf) in *S. lugdunensis*. Previous studies have shown that vWbl has four regions, including the signal peptide (S), the non-repetitive (A) region, the repeat (R) region, and the wall-associated (W) region (Nilsson et al., 2004).

A stretch of 24 amino acids in the R region of vWbl is reported to be required for interaction with vWf (Nilsson et al., 2004). An initial study investigating the *vWbl* gene in *S. lugdunensis* reported 10 sequence repeats in the R region (Nilsson et al., 2004). However, we found number of repeats in the R region varied among different isolates. In this study, we conducted an epidemiological study to determine whether the number of repeats in the R region of isolates of *S. lugdunensis* correlates with their clonality.

## Material And Methods

### Bacterial Isolates

A total of 212 *S. lugdunensis* isolates, consisting of 162 isolates of oxacillin-sensitive *S. lugdunensis* (OSSL) and 50 isolates of oxacillin-resistant *S. lugdunensis* (ORSL) were collected from different types of specimens, between 2003 and 2014, in Chang-Gung Memorial Hospital (CGMH), Linkou, as described previously (Yeh et al., 2016). Strains were classified into three groups. i.e., HA (hospital-acquired), CA (community-acquired), and Contamination (Co). Strains that were identified more than 48 hours after the admission of the patients to the hospital or isolated from long-term hospitalized patients were classified as HA infections, and strains with none of the above-mentioned features were classified as CA infections. Blood-isolated strains that were not accompanied with symptoms of bacteremia, such as prolonged fever or hypotension, were classified as contamination. Isolates were identified as *S. lugdunensis via* traditional biochemical tests and Bruker Biotyper (database 2.0) matrix-assisted laser desorption/ionization time-of-flight mass spectrometry (MALDI-TOF MS). Isolates were also assessed by multi-locus sequence typing (MLST) and staphylococcal chromosome cassette *mec* (SCC*mec*)-typing, as described previously (Kondo et al., 2007; Chassain et al., 2012). The evolution of various sequence types (STs) was analyzed using eBURST (http://eburst.mlst.net/).

### Sequence Determination of the Gene Encoding vWbl

The *vWbl* repeat region was amplified by PCR using the forward primer, vWblF (5′-TGCAACAATTCCAGATCGCG-3′) described previously (Nilsson et al., 2004), and a newly designed reverse primer, vWblR-1 (5′- GGTAACTTGACACATGCATATC-3′), which binds to an area near the region encoding the cell-wall anchoring LPXTG motif. PCR products were sequenced using the Dynamic ET Terminator Cycle Sequencing Premix kit (Amersham Biosciences) on a DNA sequencer (model no. 377; PerkinElmer^®^). The number of sequence repeats were determined from the size of the PCR product, which was based on our CGMH-118 and CGMH-131 *vWbl* gene sequencing data, as previously described (Nilsson et al., 2004). Briefly, the vWblF forward primer was located immediately upstream of the first repeat, at 149 bp, in the R region, and the vWblR-1 reverse primer was located just after 141^st^ bp in the last repeat. Each repeat was 201 bp in length.

### Sequence Comparison

Novel *vWbl* gene sequences from two *S. lugdunensis* isolates, CGMH-SL118 and CGMH-SL131, were generated and submitted to NCBI GenBank (BankIt2259998 SL118, MN412410, and BankIt2259998 SL131, MN412411). The sequences of 13 reference *S. lugdunensis* strains were downloaded from the NCBI genome database (FDAARGOS 141, FDAARGOS 143, FDAARGOS 377, HKU09, Klug93g-4, N920143, NCTC7990, NCTC12217, VISLISI 21, VISLISI 22, VISLISI 25, VISLISI 27, and VISLISI 33) and used for analyses.

### Western Blotting

To estimate the expression of vWbl, the region spanning amino acids 1113–1337 of the von Willebrand factor-binding protein was chosen as the antigen for generating the anti-vWbl antibody. This fragment was synthesized and subcloned into the pGEX-4T-AB1 vector with GST-Tag (211aa) and His-Tag (6aa) for overexpression of the antigen peptide, which was used to immunize rabbits for antibody production (ABclonal^®^). Briefly, 0.8 mM IPTG was used to induce the overexpression of vWbl (1113-1337)-GST in the strain at 37°C for 4 hours; the expressed vWbl (1113-1337)-GST was further purified using a His-Tag column. The purified peptide was administered four times to two New Zealand rabbits, and sera were collected after 94 days from the first immunization. The concentration of purified antiserum was measured to be 1.78 mg/mL (rabbit 1) and 0.37 mg/mL (rabbit 2), and the antibody was diluted by 1000-fold. Western blotting was performed as previously described (Hendrickx et al., 2007). Briefly, a 100-fold dilution of overnight bacterial culture was refreshed for 6 h, and the supernatant and pellet were collected after centrifugation at 12470 g for 5 min. The supernatant was centrifuged (12000 g, 30 min) for exoprotein condensation. The pellet was resuspended in 200 μL phosphate buffer, homogenized with liquid nitrogen, centrifuged at 18620 g for 1 h, and the total cell lysate was harvested. The protein loads for the cell lysates and exoprotein samples were equalized and then separated on 10% sodium dodecyl sulfate-polyacrylamide gel electrophoresis (SDS-PAGE). After SDS-PAGE, samples were transferred onto a polyvinylidene fluoride (PVDF) membrane and incubated with anti-vWbl antibody (1: 1000) for 10 mins, followed by incubation with a goat anti-rabbit IgG secondary antibody (1:10000, Abnova™) for 1 h. Post incubation, the sample signal was visualized using ECL (PerkinElmer^®^) chemiluminescent detection substrate.

## Results

### Number of Sequence Repeats in the R Region of *vWbl* in *S. lugdunensis* Isolates

**Figure 1:**
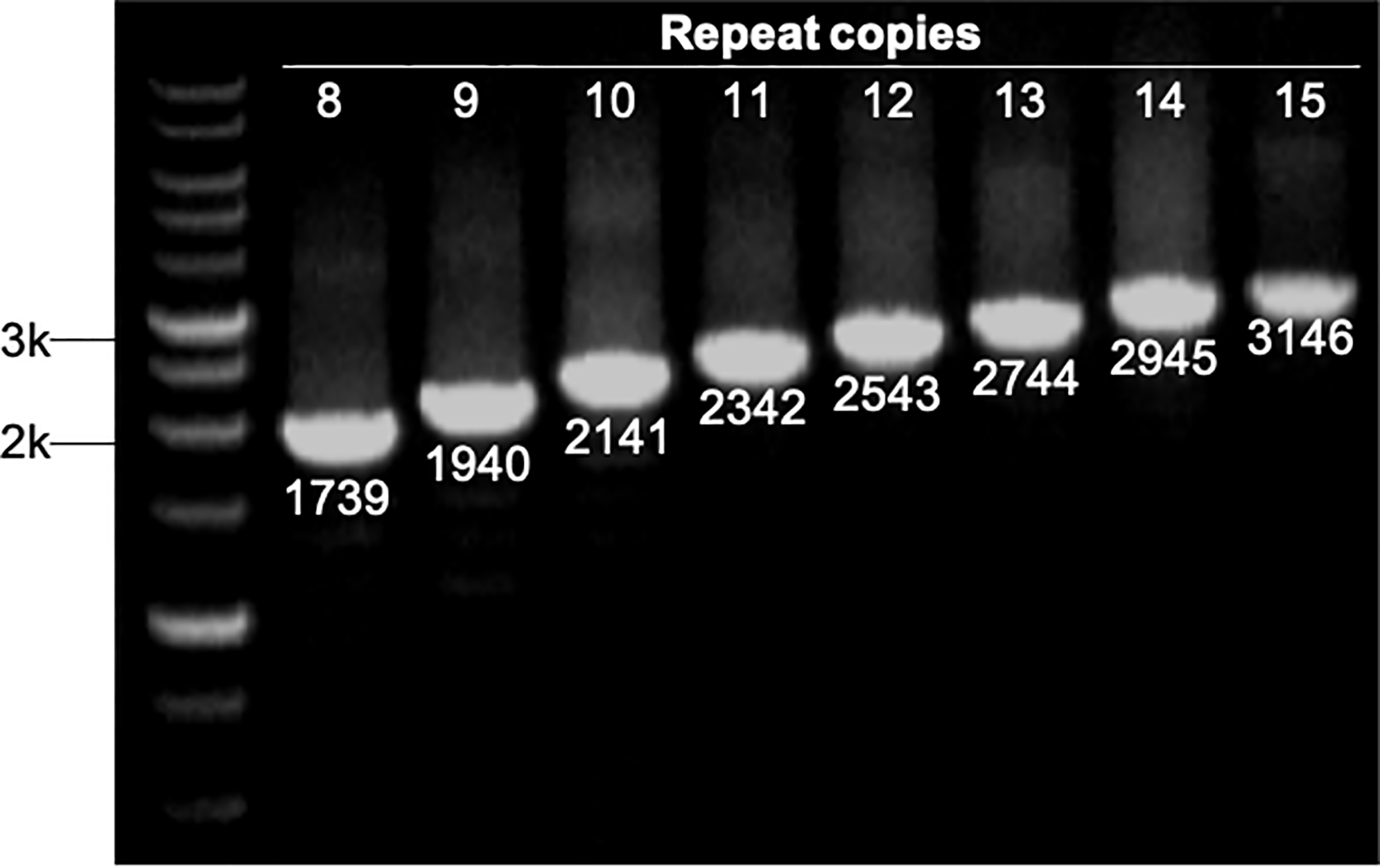
Strains with different repeat copies were examined by PCR. All PCR products were sequenced to determine their sizes, which represented different repeat copies ranged from 1739 bp (8 copies) to 3146 bp (15 copies). M represents 1 kb markers.

**Figure 2:**
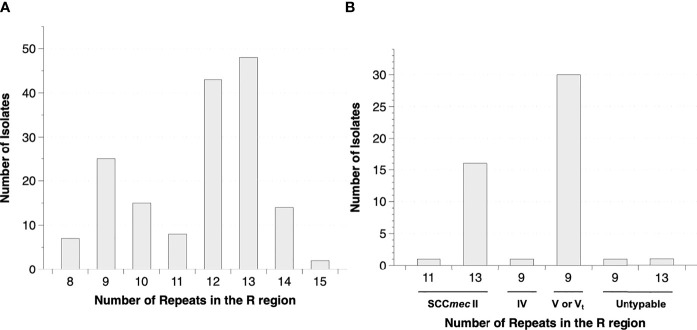
Different repeat copies distribution in **(A)** oxacillin-sensitive *Staphylococcus lugdunensis* (OSSL); **(B)** oxacillin-resistant *S. lugdunensis* (ORSL) with different SCC*mec*.

**Figure 3:**
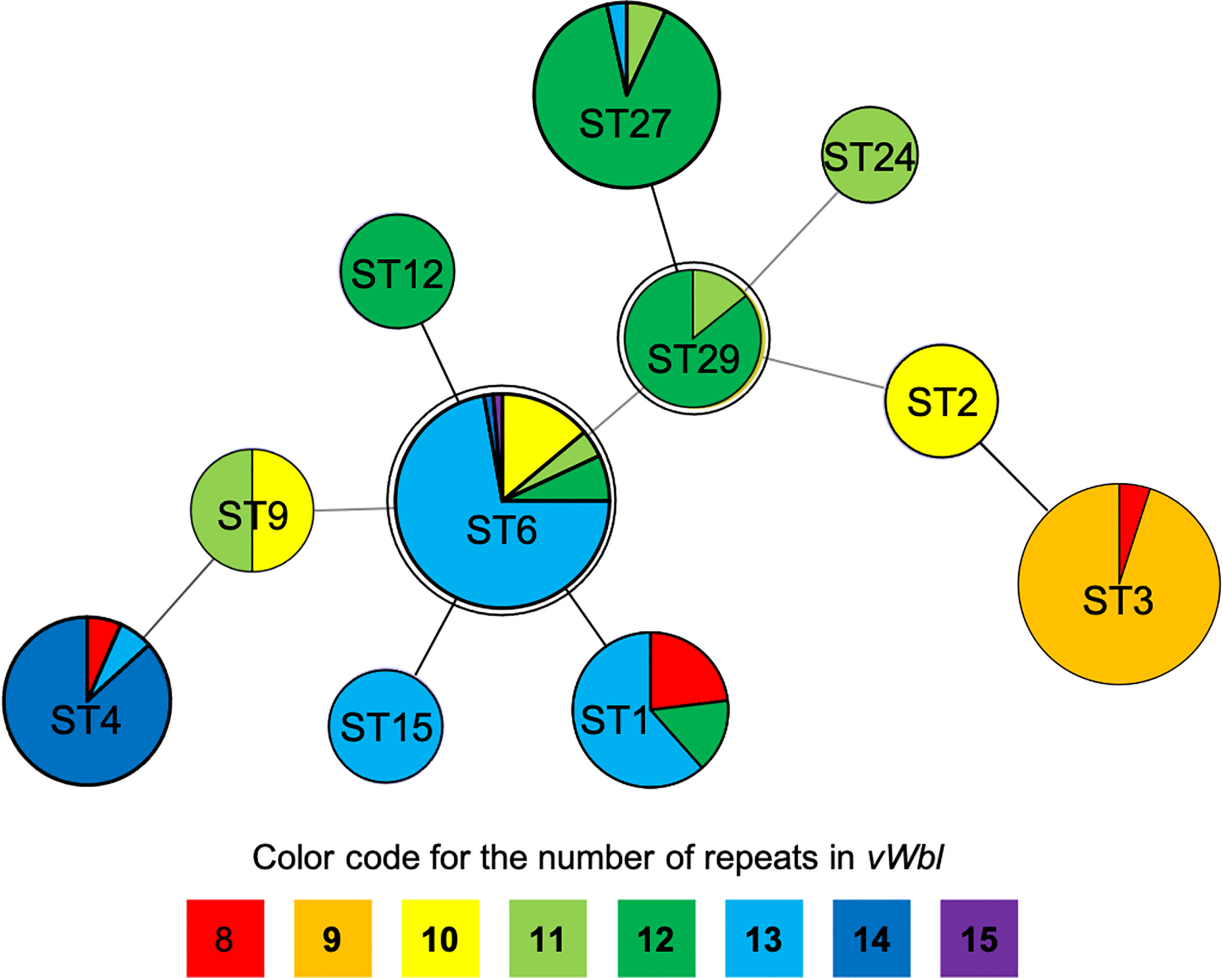
Repeat copies distribution on eBURST diagram of STs. The double circles (ST6 and ST29) represent the founders in this diagram while different colors represent specific repeats as displayed at the bottom of the phylogenetic chart.

The number of sequence repeats in the R region of *vWbl* was determined based on the size of the sequenced PCR products, which ranged from 1739 bp (8 repeats) to 3146 bp (15 repeats) among the various isolates (**Figure 1**). Isolates from blood, deep tissue, pus, and wounds with 9, 12, or 13 repeats were the most abundant (**Table 1A**). We further evaluated the correlation of infection types with the repeat copies; most of the HA (hospital-acquired) and CA (community-acquired) isolates belonged to 9, 12, and 13 copies (**Table 1B**). Among the 162 OSSL isolates, those with 9, 12, or 13 repeats were predominant (**Figure 2A**). Four of these isolates were obtained from blood samples of patients with endocarditis, and these isolates had 8, 10, 12, or 13 repeats. Among the 50 ORSL isolates, 32 had 9 repeats, and 17 had 13 repeats (**Figure 2B**). Most (16/17) SCC*mec* type II isolates had 13 repeats, and all (30) SCC*mec* type V or V_T_ isolates, belonging to ST3, had 9 repeats (**Figure 2B**). Most isolates with 12 (60.5%, 26/43), 13 (80%, 52/65), or 14 (92.8%, 13/14) repeats belonged to ST27, ST6, and ST4, respectively, and most ST3 isolates contained 9 repeats (**Table 2** and **Figure 3**). To determine the correlation between ST and repeat copies, we merged the repeat copy distribution with the eBURST diagram of the STs to represent the evolutionary relationships among the STs. (**Figure 3**). Most of the repeat copy numbers and their neighboring repeat copy numbers belonged to the same ST or neighboring STs, that is they belonged to the two founder STs, ST6 and ST29, and the STs connected to them. Isolates from ST12, ST27, ST24, and ST29 contained similar number of repeats (11 or 12 repeats) in the R region and exhibited a close evolutionary relationship (**Figure 3**). A similar observation was made for ST6, ST15, and ST1 isolates, which contained 13 repeats (**Figure 3**).

**Table 1A:** Number of repeats in the R region of *vWbl* in isolates obtained from different types of specimens and infection types. A. Repeat number distribution among various specimens types.

Number of repeats	Type of specimens* and number of isolates (%)														Total
	AB	AM	AS	B	BF	CSF	CX	DTS	OTH	PL	Pus	SY	TS	WD	
8	0	0	0	2 (1.9)	0	0	0	2 (8)	0	0	3 (10.3)	0	0	0	7 (3.3)
9	1 (25)	0	2 (33.3)	32 (29.6)	1 (33.3)	1 (100)	1 (33.3)	9 (36)	2 (20)	0	3 (10.3)	0	0	5 (27.80)	57 (26.9)
10	1 (25)	1 (100)	0	5 (4.6)	1 (33.3)	0	0	1 (4)	2 (20)	0	3 (10.3)	1 (50)	0	0	15 (7.1)
11	0	0	0	3 (2.8)	0	0	0	1 (4)	0	1 (100)	2 (6.9)	0	0	2 (11.1)	9 (4.2)
12	1 (25)	0	1 (16.6)	20 (18.5)	1 (33.3)	0	0	6 (24)	5 (50)	0	4 (13.8)	0	1 (100)	4 (22.2)	43 (20.3)
13	1 (25)	0	1 (16.6)	37 (34.3)	0	0	1 (33.3)	6 (24)	1 (10)	0	10 (34.5)	1 (50)	0	7 (38.9)	65 (30.7)
14	0	0	2 (33.3)	8 (7.4)	0	0	1 (33.3)	0	0	0	3 (10.3)	0	0	0	14 (6.6)
15	0	0	0	1 (0.9)	0	0	0	0	0	0	1 (3.4)	0	0	0	2 (0.9)
Total	4	1	6	108	3	1	3	25	10	1	29	2	1	18	212

*AB, abscess; AM, amniotic fluid; AS, ascites; B, blood; BF, body fluid; CSF, cerebrospinal fluid; CX, endocervix discharge; DTS, deep tissue; OTH, others; PL, pleural effusion; SY, synovial fluid; TS, tissue; WD, wound.

**Table 1B:** Repeat number distribution among different infection types.

Infection Type	Number of repeats								
	8	9	10	11	12	13	14	15	Total
HA^*^	1	28	8	1	14	21	6	0	79
CA	6	16	4	5	22	24	3	1	81
Co	0	13	3	3	7	20	5	1	52
Total	7	57	15	9	43	65	14	2	212

*HA, hospital acquired,

CA, community acquired.

Co, contamination.

**Table 2:** Number of repeats in the R region of *vWbl* in the various MLST types.

MLST	Number of repeats								Total
	8	9	10	11	12	13	14	15	
ST1	3	–	–	–	2	8	–	–	13
ST2	–	–	3	–	–	–	–	–	3
ST3	3	57	–	–	–	–	–	–	60
ST4	1	–	–	–	–	1	13	–	15
ST6	–	–	10	3	5	52	1	1	72
ST9	–	–	2	2	–	–	–	–	4
ST12	–	–	–	–	4	–	–	–	4
ST15	–	–	–	–	–	3	–	–	3
ST24	–	–	–	1	–	–	–	–	1
ST27	–	–	–	2	26	1	–	–	29
ST29	–	–	–	1	6	–	–	–	7
Untypable	–	–	–	–	–	–	–	1	1
Total	7	57	15	9	43	65	14	2	212

### Stop Codon Present in Third Repeat of Isolates With Nine Repeats

**Figure 4:**
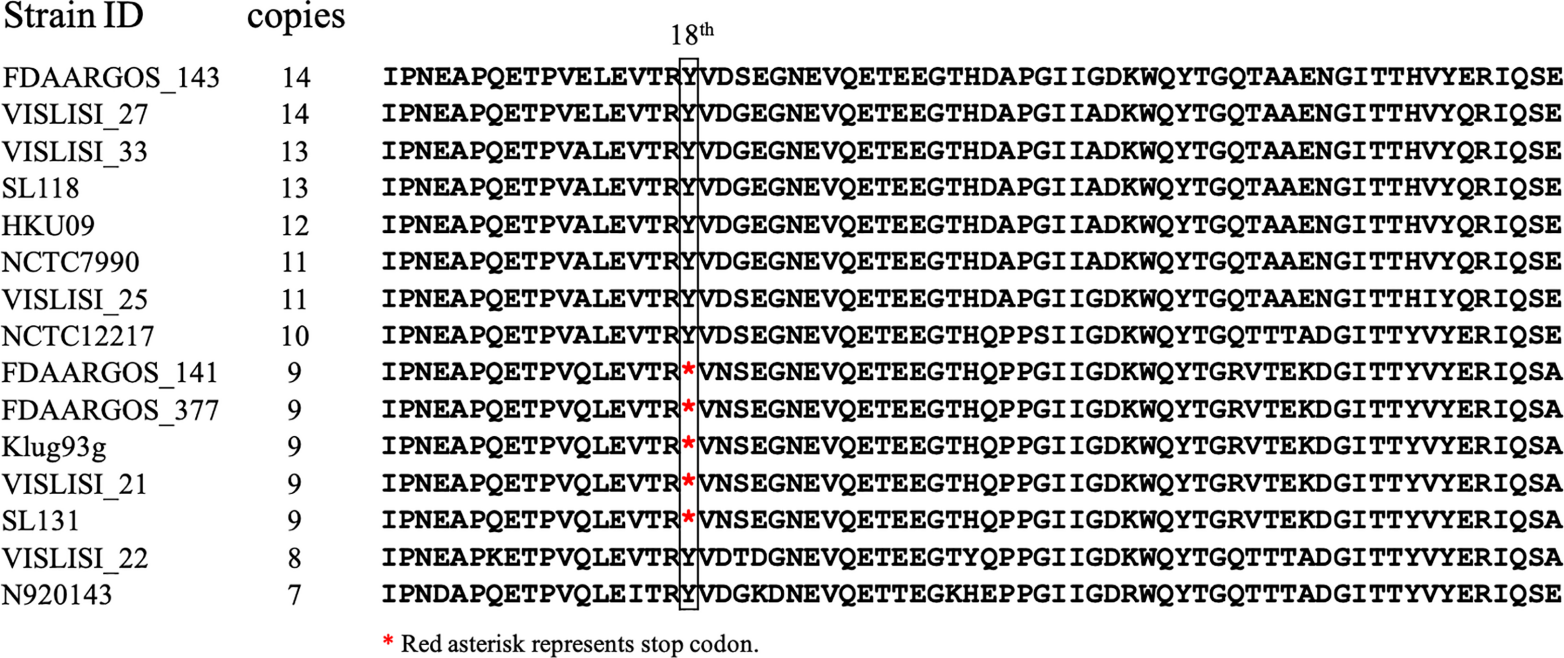
Amino acid sequence comparison of the third repeat in different vWbl copies. vWbl third repeat amino acid sequence from 13 reference strains and two clinical isolates (CGMH-SL118 and CGMH-SL131) were compared as shown. The red asterisk represents the stop codon.

To investigate the differences among different repeat numbers, the sequences of the all repeats of two clinical isolates [CGMH-SL118 (13 repeats) and CGMH-SL131 (9 repeats)] and 13 reference strains were analyzed, and we found that the 18^th^ codon in the third repeat of isolates with nine repeats is a stop codon (TAA), which is not present in isolates with numbers of repeats other than 9 (**Figure 4** and **Table 3**). All OSSL (25) and ORSL (32) isolates with 9 repeats had a stop codon (TAA) at the 18^th^ codon in the third repeat (**Table 3**).

**Table 3:** Nucleotide sequences of the 18^th^ codon in the 3^rd^ repeat of various isolates with nine repeats in the R region of *vWbl*.

		TAA^*^	TAT
OSSL (25)		25	0
ORSL (32)	SCC*mec* IV (1)	1	0
	SCC*mec* V (27)	27	
	SCC*mec* V_t_ (3)	3	
	Untypable (1)	1	
Total (57)		57 (100%)	0

^*^Stop codon; values shown are the number of isolates.

### Western Blot Analysis of the Expression of vWbl With Different Number of Repeats

**Figure 5:**
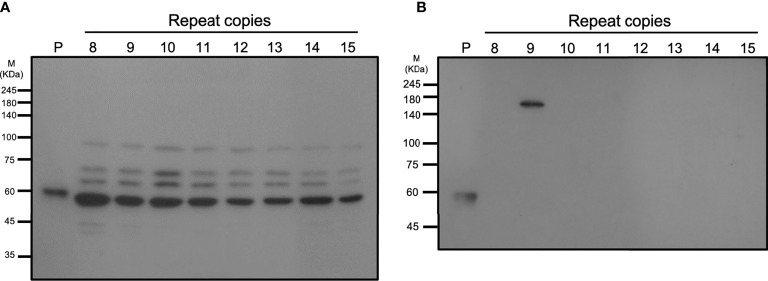
Western blotting analysis of different strains with various repeat copies. **(A)** shows the total cell lysates, and **(B)** shows the exoproteins of different strains.

A specific anti-vWbl antibody was generated to estimate the expression of vWbl (**Figure 5**). Isolates with different copy numbers all express a similar amount of vWbl (**Figure 5A**). To determine the expression of the LPXTG motif in the C-terminal, exoproteins of isolates with different copy numbers were analyzed by western blotting, and only those with nine repeats expressed the exoprotein signal (**Figure 5B**).

## Discussion

*S. lugdunensis* is a life-threatening pathogen and causes prosthetic device-associated endocarditis, which has a high mortality rate (Flores Umanzor et al., 2016; Ishiekwene et al., 2017). Previous studies have shown that *S. lugdunensis* can interact with platelets through binding of the surface protein vWbl to vWf (Nilsson et al., 2004). The R region of the *vWbl* gene has been shown to contain 10 copies of a 201-bp sequence (Nilsson et al., 2004). However, we found variable copy numbers of repeats in the R region among different isolates. In this study, our data suggested that *S. lugdunensis* isolates of different SCC*mec* and MLST types, obtained from different specimens, have a relatively unique number of repeats in the R region of *vWbl.*

Our results suggest that isolation sources and types do not appear to be correlated with the repeat copies (**Table 1A**), and different isolates had a unique number of repeats in the R region of *vWbl*, which were correlated with strain molecular types. Most of the repeat copies of the isolation source and types were distributed in 9, 12, and 13 copies, which was consistent with the distribution of strain molecular types. Our phylogenetic analysis revealed that ST29 and ST6 were the founder STs. Interestingly, most STs that extended from these founder STs showed a similar or a smaller repeat number, suggesting that a lower number of repeats may be accompanied with the evolution of STs. Furthermore, considering the sequence conservation of the first and last repeats in each copy, the decreased number of repeats may come from the recombination and removal of repeat copies.

A previous animal study showed that *vWbl* null mutants display reduced virulence compared to wild-type *S. lugdunensis* (Heilbronner et al., 2013), and further studies also considered vWbl as a virulence factor (Didi et al., 2014; Giormezis et al., 2015). This potential virulence factor is an LPXTG-motif-containing surface protein, which is recognized by sortase A and anchors it in the cell wall (Mazmanian et al., 1999). Our sequence analyses revealed that all isolates with nine repeats had a stop codon at the 18^th^ codon in the third repeat (**Table 3**), a feature that resulted in the loss of the LPXTG motif at the C-terminal end, suggesting that isolates with nine repeats in the R region of *vWbl* may not anchor into the correct location in the cell wall. Further western blot analysis of the exoprotein fraction showed that only vWbl proteins with nine copies of the repeat sequence were exported outside the cell, and the molecular weight was equivalent to the predicted size (**Figure 5B**), which was consistent with our hypothesis that the LPXTG motif has been lost.

Previous studies investigating infectious bacteremia have led to the formulation of the neutrophil extracellular trap (NET) theory (Wartha et al., 2007). Infectious bacteremia is usually accompanied with pathogen dissemination (Moreillon et al., 2002). To restrict pathogen dissemination, platelets interact with pathogens, activate thrombosis, and develop NETs (Jung et al., 2015), which have been found to capture pathogens (Jung et al., 2015). Since all SCC*mec* V or V_t_ isolates of *S. lugdunensis* had a stop codon at the 18^th^ codon in the third repeat of the R region, vWbl in these isolates cannot be maintained at the cell wall, are exported outside the cell, and thus these isolates may not be captured by NETs. Our previous epidemiological investigation also showed that isolates of *S. lugdunensis* SCC*mec* V or V_t_ disseminate to a higher degree (Lin et al., 2015; Yeh et al., 2015; Yen et al., 2016).

As the whole cell lysate of each isolate showed a similar pattern to that in the western blot, we assume that some protease may have been released during our protein extraction process, leading to this result. A previous study of vWbl indicated that a non-repetitive region (A-region) is in front of the R-region (Nilsson et al., 2004), which showed sequence conservation among vWbl with any repeat copies, suggesting it is possible that the cleavage occurred on the A-region and caused a similar pattern of each vWbl with any repeat copies in our western blot analysis (**Figure 5A**). In addition, some of the vWbl may have escaped from the cleavage, which also caused the vWbl with nine copies to be exported outside the bacterial cell and detected in our western blot result (**Figure 5B**). However, further investigations are needed to prove the hypothesis.

Our data showed that repeat copies of vWbl were highly correlated with the strain molecular types; however, few references were available for comparison with our analysis results and no particular types of severe infections were found in strains with different vWbl repeats in this study. Various molecular types may have diverse virulence mechanisms (in addition to vWbl); thus, it is difficult to conclude that the various repeat copies of vWbl in specific molecular types is responsible for the strains’ virulence or pathogenicity. Therefore, our results may be a local area phenomenon as all isolates were collected from a single medical center. Further investigation is needed to collect isolates from more than one location and investigate their virulence or pathogenicity. In conclusion, our data showed that isolates with a certain number of repeats belong to specific molecular types. Our results also showed that strains, such as SCC*mec* V or V_t_, might produce nonfunctional vWbl because of the presence of a stop codon in the repeat region. The molecular mechanisms underlying the evolution of different strains with a unique number of repeats in the R region of *vWbl* remain to be investigated.

## Data Availability Statement

The datasets presented in this study can be found in online repositories. The names of the repository/repositories and accession number(s) can be found below: https://www.ncbi.nlm.nih.gov/nuccore/MN412410, MN412410 https://www.ncbi.nlm.nih.gov/nuccore/MN412411, MN412411.

## Ethics Statement

This study was approved by the Ethics Committee of Linkou Chang-Gung Memorial Hospital. Because this study only experimented on bacteria and did not affect the patients adversely, the Review Board agreed the usage of the requested bacterial materials from Linkou Chang-Gung Memorial Hospital bacterial storage. Two of the ethics committee approved documents are included in the additional files. The ethics committee waived the requirement of written informed consent for participation.

## Author Contributions

J-JL, L-CL, and C-WC designed the study and wrote the manuscript. L-CL and S-CC conducted the experimental work. L-CL and J-JL analyzed the data. All authors contributed to the article and approved the submitted version.

## Funding

This work was supported by grants from the Ministry of Science and Technology, Taiwan (MOST 110-2320-B-182A-006-MY3, MOST 110-2811-B-182A-505).

## Conflict of Interest

The authors declare that the research was conducted in the absence of any commercial or financial relationships that could be construed as a potential conflict of interest.

## Publisher’s Note

All claims expressed in this article are solely those of the authors and do not necessarily represent those of their affiliated organizations, or those of the publisher, the editors and the reviewers. Any product that may be evaluated in this article, or claim that may be made by its manufacturer, is not guaranteed or endorsed by the publisher.

## References

[B1] Bjerketorp J. Nilsson M. Ljungh A. Flock J. I. Jacobsson K. Frykberg L. (2002). A Novel Von Willebrand Factor Binding Protein Expressed by Staphylococcus Aureus. Microbiology 148, 2037–2044. doi: 10.1099/00221287-148-7-2037 12101292

[B2] Chassain B. Lemee L. Didi J. Thiberge J. M. Brisse S. Pons J. L.. (2012). Multilocus Sequence Typing Analysis of Staphylococcus Lugdunensis Implies a Clonal Population Structure. J. Clin. Microbiol. 50, 3003–3009. doi: 10.1128/JCM.00988-12 22785196 PMC3421835

[B3] Didi J. Lemee L. Gibert L. Pons J. L. Pestel-Caron M. (2014). Multi-Virulence-Locus Sequence Typing of Staphylococcus Lugdunensis Generates Results Consistent With a Clonal Population Structure and is Reliable for Epidemiological Typing. J. Clin. Microbiol. 52, 3624–3632. doi: 10.1128/JCM.01370-14 25078912 PMC4187764

[B4] Douiri N. Hansmann Y. Lefebvre N. Riegel P. Martin M. Baldeyrou M.. (2016). Staphylococcus Lugdunensis: A Virulent Pathogen Causing Bone and Joint Infections. Clin. Microbiol. Infect. 22, 747–748. doi: 10.1016/j.cmi.2016.05.031 27297318

[B5] Fernández-Rufete A. García-Vázquez E. Hernández-Torres A. Canteras M. Ruiz J. Gómez J. (2012). Coagulase-Negative Staphylococcus Bacteraemia: Prognosis Factors and Influence of Antibiotic Treatment. Rev. Esp Quimioter 25, 199–205. 22987266

[B6] Flores Umanzor E. J. San Antonio R. Jimenez Britez G. Caldentey G. (2016). Staphylococcus Lugdunensis: An Unusual and Aggressive Cause of Infective Endocarditis. BMJ Case Rep. Aug 23, 2016. doi: 10.1136/bcr-2016-217156 PMC5015169 27555044

[B7] Giormezis N. Kolonitsiou F. Makri A. Vogiatzi A. Christofidou M. Anastassiou E. D.. (2015). Virulence Factors Among Staphylococcus Lugdunensis are Associated With Infection Sites and Clonal Spread. Eur. J. Clin. Microbiol. Infect. Dis. 34, 773–778. doi: 10.1007/s10096-014-2291-8 25471196

[B8] Heilbronner S. Hanses F. Monk I. R. Speziale P. Foster T. J. (2013). Sortase A Promotes Virulence in Experimental Staphylococcus Lugdunensis Endocarditis. Microbiology 159, 2141–2152. doi: 10.1099/mic.0.070292-0 23943787

[B9] Hendrickx A. P. van Wamel W. J. Posthuma G. Bonten M. J. Willems R. J. (2007). Five Genes Encoding Surface-Exposed LPXTG Proteins are Enriched in Hospital-Adapted Enterococcus Faecium Clonal Complex 17 Isolates. J. Bacteriol. 189, 8321–8332. doi: 10.1128/JB.00664-07 17873043 PMC2168695

[B10] Ishiekwene C. Ghitan M. Kuhn-Basti M. Chapnick E. Lin Y. S. (2017). Staphylococcus Lugdunensis Endocarditis With Destruction of the Ventricular Septum and Multiple Native Valves. IDCases 7, 14–15. doi: 10.1016/j.idcr.2016.10.011 27920984 PMC5133647

[B11] Jung C. J. Yeh C. Y. Hsu R. B. Lee C. M. Shun C. T. Chia J. S. (2015). Endocarditis Pathogen Promotes Vegetation Formation by Inducing Intravascular Neutrophil Extracellular Traps Through Activated Platelets. Circ 131, 571–581. doi: 10.1161/CIRCULATIONAHA.114.011432 25527699

[B12] Kondo Y. Ito T. Ma X. X. Watanabe S. Kreiswirth B. N. Etienne J.. (2007). Combination of Multiplex PCRs for Staphylococcal Cassette Chromosome Mec Type Assignment: Rapid Identification System for Mec, Ccr, and Major Differences in Junkyard Regions. Antimicrob. Agents Chemother. 51, 264–274. doi: 10.1128/AAC.00165-06 17043114 PMC1797693

[B13] Lin J. F. Cheng C. W. Kuo A. J. Liu T. P. Yang C. C. Huang C. T.. (2015). Clinical Experience and Microbiologic Characteristics of Invasive Staphylococcus Lugdunensis Infection in a Tertiary Center in Northern Taiwan. J. Microbiol. Immunol. Infect. 48, 406–412. doi: 10.1016/j.jmii.2013.12.010 24529852

[B14] Liu P. Y. Huang Y. F. Tang C. W. Chen Y. Y. Hsieh K. S. Ger L. P.. (2010). Staphylococcus Lugdunensis Infective Endocarditis: A Literature Review and Analysis of Risk Factors. J. Microbiol. Immunol. Infect. 43, 478–484. doi: 10.1016/S1684-1182(10)60074-6 21195974

[B15] Mazmanian S. K. Liu G. Ton-That H. Schneewind O. (1999). Staphylococcus Aureus Sortase, an Enzyme That Anchors Surface Proteins to the Cell Wall. Science 285, 760–763. doi: 10.1126/science.285.5428.760 10427003

[B16] Molina J. Peñuela I. Lepe J. A. Gutiérrez-Pizarraya A. Gómez M. J. García-Cabrera E.. (2013). Mortality and Hospital Stay Related to Coagulase-Negative Staphylococci Bacteremia in non-Critical Patients. J. Infect. 66, 155–162. doi: 10.1016/j.jinf.2012.10.021 23103291

[B17] Moreillon P. Que Y. A. Bayer A. S. (2002). Pathogenesis of Streptococcal and Staphylococcal Endocarditis. Infect. Dis. Clin. North Am. 16, 297–318. doi: 10.1016/S0891-5520(01)00009-5 12092474

[B18] Nilsson M. Bjerketorp J. Wiebensjo A. Ljungh A. Frykberg L. Guss B. (2004). A Von Willebrand Factor-Binding Protein From Staphylococcus Lugdunensis. FEMS Microbiol. Lett. 234, 155–161. doi: 10.1111/j.1574-6968.2004.tb09527.x 15109734

[B19] Wartha F. Beiter K. Normark S. Henriques-Normark B. (2007). Neutrophil Extracellular Traps: Casting the NET Over Pathogenesis. Curr. Opin. Microbiol. 10, 52–56. doi: 10.1016/j.mib.2006.12.005 17208512

[B20] Wu A. B. Wang M. C. Tseng C. C. Lin W. H. Teng C. H. Huang A. H.. (2011). Clinical and Microbiological Characteristics of Community-Acquired Staphylococcus Lugdunensis Infections in Southern Taiwan. J. Clin. Microbiol. 49, 3015–3018. doi: 10.1128/JCM.01138-11 21697317 PMC3147778

[B21] Yeh C. F. Chang S. C. Cheng C. W. Lin J. F. Liu T. P. Lu J. J. (2016). Clinical Features, Outcomes, and Molecular Characteristics of Community- and Health Care-Associated Staphylococcus Lugdunensis Infections. J. Clin. Microbiol. 54, 2051–2057. doi: 10.1128/JCM.00847-16 27225402 PMC4963507

[B22] Yeh C. F. Liu T. P. Cheng C. W. Chang S. C. Lee M. H. Lu J. J. (2015). Molecular Characteristics of Disease-Causing and Commensal Staphylococcus Lugdunensis Isolates From 2003 to 2013 at a Tertiary Hospital in Taiwan. PloS One 10, e0134859. doi: 10.1371/journal.pone.0134859 26248332 PMC4527845

[B23] Yen T. Y. Sung Y. J. Lin H. C. Peng C. T. Tien N. Hwang K. P.. (2016). Emergence of Oxacillin-Resistant Staphylococcus Lugdunensis Carrying Staphylococcal Cassette Chromosome Mec Type V in Central Taiwan. J. Microbiol. Immunol. Infect. 49, 885–891. doi: 10.1016/j.jmii.2014.11.018 25648670

